# Supplementary material for: Risk of Developing Metabolic Syndrome Is Affected by Length of Daily Siesta: Results from a Prospective Cohort Study
Source: Nutrients. 2021 Nov 22;13(11):4182. doi: 10.3390/nu13114182 (PMC8619148; doi:10.3390/nu13114182)
Supplement: Supplementary file 1 [file nutrients-13-04182-s001.zip › supplementary.pdf]

Table S1. Sensitivity analysis: Adjusted odd ratios (aOR)* and 95% confidence intervals (CI) for the development of the Metabolic Syndrome according to frequency of siesta habit in the SUN cohort

	**Any siesta**		**Short siesta (≤30mins)**		**Long siesta (>30mins)**	
**Never**	N	3719	N	5812	N	5968
	Cases	120	Cases	232	Cases	213
	aOR (95%CI)	Ref.	aOR (95%CI)	Ref.	aOR (95%CI)	Ref.
**2 days a week**	N	1884	N	1017	N	1967
	Cases	65	Cases	35	Cases	79
	aOR (95%CI)	1.00 (0.72-1.38)	aOR (95%CI)	0.92 (0.63-1.34)	aOR (95%CI)	1.12 (0.85-1.48)
**5 days a week**	N	527	N	1337	N	290
	Cases	18	Cases	54	Cases	13
	aOR (95%CI)	0.95 (0.56-1.60)	aOR (95%CI)	0.97 (0.71-1.33)	aOR (95%CI)	1.45 (0.79-2.76)
**7 days a week**	N	3031	N	995	N	936
	Cases	171	Cases	53	Cases	69
	aOR (95%CI)	1.27 (0.98-1.64)	aOR (95%CI)	1.00 (0.72-1.38)	aOR (95%CI)	1.43 (1.05-1.95)

*Adjusted for sex, age, years of university, year of entry into cohort, working hours, lunch at home, hours of night-time sleep, daily TV, smoking pack-years, daily alcohol intake, total daily energy intake, daily coffee intake, Mediterranean Diet Score, special diets, physical activity, social time, prevalent cardiovascular disease, prevalent cancer, prevalent depression, prior history of insomnia, obstructive sleep apnea, snoring, weight gain prior to recruitment, tendency to stress.
